# Familial partial lipodystrophy resulting from loss-of-function PPARγ pathogenic variants: phenotypic, clinical, and genetic features

**DOI:** 10.3389/fendo.2024.1394102

**Published:** 2024-09-27

**Authors:** Reivla Marques Vasconcelos Soares, Monique Alvares da Silva, Julliane Tamara Araújo de Melo Campos, Josivan Gomes Lima

**Affiliations:** ^1^ Department of Clinical Medicine, Hospital Universitário Onofre Lopes (HUOL), Federal University of Rio Grande do Norte (UFRN), Natal, RN, Brazil; ^2^ Molecular Biology and Genomics Laboratory, Federal University of Rio Grande do Norte (UFRN), Natal, RN, Brazil; ^3^ Department of Morphology (DMOR), Federal University of Rio Grande do Norte (UFRN), Natal, RN, Brazil

**Keywords:** PPAR gamma, adipose tissue, genetic lipodystrophy, insulin resistance, diabetes mellitus

## Abstract

The *PPARG* gene encodes a member of a nuclear receptor superfamily known as peroxisome proliferator-activated gamma (PPARγ). PPARγ plays an essential role in adipogenesis, stimulating the differentiation of preadipocytes into adipocytes. Loss-of-function pathogenic variants in *PPARG* reduce the activity of the PPARγ receptor and can lead to severe metabolic consequences associated with familial partial lipodystrophy type 3 (FPLD3). This review focuses on recent scientific data related to FPLD3, including the role of PPARγ in adipose tissue metabolism and the phenotypic and clinical consequences of loss-of-function variants in the *PPARG* gene. The clinical features of 41 *PPARG* pathogenic variants associated with FPLD3 patients were reviewed, highlighting the genetic and clinical heterogeneity observed among 91 patients. Most of them were female, and the average age at the onset and diagnosis of lipoatrophy was 21 years and 33 years, respectively. Considering the metabolic profile, hypertriglyceridemia (91.9% of cases), diabetes (77%), hypertension (59.5%), polycystic ovary syndrome (58.2% of women), and metabolic-dysfunction-associated fatty liver disease (87,5%). We also discuss the current treatment for FPLD3. This review provides new data concerning the genetic and clinical heterogeneity in FPLD3 and highlights the importance of further understanding the genetics of this rare disease.

## 1 Introduction

Lipodystrophies are rare conditions resulting from disturbances in adipogenesis or lipid storage, culminating in a loss of adipose tissue without nutritional restriction or catabolic state. The estimated prevalence of these syndromes ranges from 1.3-4.7 cases per million (1). It can be classified based on etiology (congenital or acquired) or the extent of lipoatrophy (partial or generalized). Based on these classifications, there are four groups of lipodystrophies: Congenital Generalized Lipodystrophy (CGL), Familial Partial Lipodystrophy (FPLD), Acquired Generalized Lipodystrophy (AGL), and Acquired Partial Lipodystrophy (APL) (2). Acquired lipodystrophies are generally associated with HIV infection and its treatment or with autoimmune diseases (3).

Among the genetic lipodystrophies, CGL has autosomal recessive inheritance, characterized by an almost complete absence of subcutaneous white adipose tissue (sWAT). Patients usually have less than 6% total body fat (4). It is considered an ultra-rare disease with a prevalence of 0.96/million, with around 500 cases described in the literature (5). In some countries, such as Brazil, the CGL prevalence is exceptionally high (32.3/million inhabitants) (6). The scarcity of adipose tissue in these patients leads to serious metabolic consequences such as severe hypertriglyceridemia (HTG), diabetes, liver cirrhosis due to steatosis, and a higher predisposition to infections. These complications reduce life expectancy by 35 years for the affected patients (7).

FPLD was initially described in 1970 and is the most common form of genetic lipodystrophy, with an estimated prevalence of 1.7-2.8 cases/million (8). This disorder results from a selective loss of adipose tissue, usually affecting the buttocks and lower limbs, with fat accumulation in other regions, such as the abdomen and neck. The relative deficiency of adipose tissue and its lipid storage capacity impairment causes metabolic consequences such as HTG, insulin resistance (IR), diabetes, non-alcoholic hepatic steatosis, hypertension, and atherosclerosis (8, 9).

FPLD has considerable genetic and phenotypic variability and can be classified into different types based on specific DNA changes. FPLD type 1 - or Köbberling syndrome - is characterized by a loss of adipose tissue concentrated in the lower limbs, but no specific genes involved have been described. FPLD type 2 – or Dunnigan’s syndrome – results from mutations in the *LMNA* gene, responsible for encoding laminas A and C, whose mutation causes cell damage and premature apoptosis of adipocytes. Type 3 FPLD has been described as associated with mutations in the *PPARG* gene, which is involved in adipogenesis. FPLD2 and FPLD3 account for almost 50% of partial lipodystrophy cases (10). FPLD2 has been reported in over 500 patients, while FPLD3 has been documented in around 20 affected families (8). Type 4 FPLD occurs due to heterozygous pathogenic variants in the *PLIN1* gene, responsible for encoding the lipid droplet-associated perilipin-1 protein (9). FPLD type 5 results from a homozygous variant in the *CIDEC* gene, involving the formation of a unique lipid droplet in white adipose cells (9). Initiated in adulthood, FPLD type 6 results from variants of LIPE, which encodes hormone-sensitive lipase (HSL). The HSL hydrolyzes adipocyte triglycerides, providing free fatty acids (FFA) and glycerol. Other types of FPLD are caused by pathogenic variants in *MFN2* and *AKT2* genes (9).

Autosomal dominant variants cause *PPARG* loss-of-function and consequent reduction in its receptor activity, leading to a severe metabolic phenotype characteristic of FPLD3 (9). These data reinforce the role of PPARγ as a fundamental regulator in adipose tissue metabolism.

This review highlights the most recent scientific evidence on FPLD3, including the role of PPARγ in adipose tissue metabolism and the phenotypic consequences of loss-of-function variants in the *PPARG* gene, emphasizing the genetic and clinical heterogeneity observed among FPLD3 patients.

## 2 PPARG and adipose tissue

The *PPARG* (peroxisome proliferator-activated receptor) gene located on the short arm of chromosome 3 (3p25.2) encodes a member of a superfamily of nuclear receptors called peroxisome proliferator-activated receptors that comprise three isoforms (PPARα, PPARγ, and PPARδ) with distinct tissue distribution and physiological roles (1). The PPARγ functions as a ligand-activated transcription factor, and it is expressed in white and brown adipose tissues (WAT and BAT, respectively), the large intestine, and the spleen. Nevertheless, its expression is higher in adipose tissue, which plays a central role in adipocyte differentiation and function (11). The focus on PPARγ as a master in adipose tissue metabolism began in the 1990s after the discovery of thiazolidinediones and their power to induce adipocyte differentiation and improve insulin sensitivity (12).

## 3 Molecular insights of PPARγ

**Figure 1:**
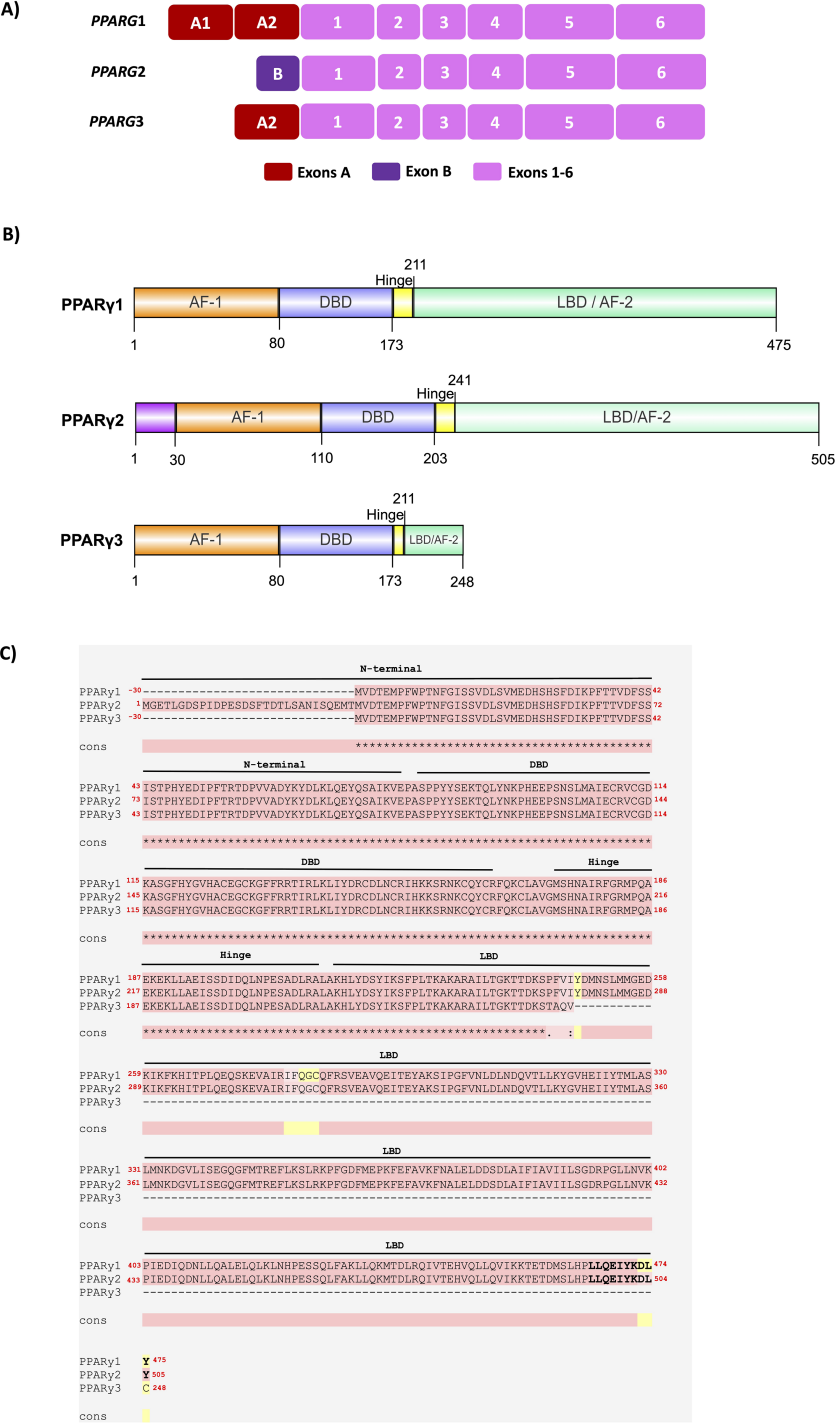
Schematic representation of the main human *PPARG* transcripts and its PPARγ protein isoforms. **(A)** The structure of the *PPARG1, 2, and 3* transcripts is highlighted, showing exons 1 to 6, common to all PPARγ transcripts, while *PPARG2* has the additional exon B and encodes the canonical and dominant PPARγ2 isoform. *PPARG1* also presents exons A1 and A2, while *PPARG3* also presents the A2 exon. **(B)** PPARγ1 has 475 amino acids and is expressed at low levels in adipose tissue, skeletal muscle, macrophages, and epithelium from the colon. PPARγ2 presents 30 additional amino acids (magenta) and is mainly found in WAT, BAT, and the liver. PPARγ3 has 248 aa and has higher expression levels in macrophages, adipose tissue, and large intestine epithelium. The main PPARγ isoforms 1, 2, and 3 are composed of 4 functional domains: N-terminus domain AF-1 (orange), DNA Binding Domain – DBD (blue), Hinge (yellow), and Ligand Binding Domain – LBD (green) in the C-terminus. The AF-1 domain and the Hinge region are poorly conserved, while the DBD, LBD, and AF-2 domains are highly conserved. The image was made using IBS 2.0 software. **(C)** Protein sequence alignment of the *PP*ARγ1, 2, and 3 isoforms. PPARγ isoform sequences were aligned via T-Coffee. Pink represents identical alignments; yellow corresponds to similar alignments; and green regions show different alignments. ∗ corresponds to an equal match. Cons: consensus sequence. The PPARγ sequences used were: PPARγ1 (NM_001354666; NP_001341595.2), PPARγ2 (NM_015869.5; NP_056953.2), and PPARγ3 (NM_001330615.4; NP_001317544.2).

*PPARG* gene has sixteen *PPARG* splicing variants in humans, according to the RefSeq database from the NCBI (National Center for Biotechnology Information). They are produced by the differential combination of alternative promoters. The three main isoforms of PPARγ in humans are PPARγ1, PPARγ2, and PPARγ3 (13). The PPARγ1 (NM_001354666; NP_001341595.2) contains 475 amino acids and is expressed at low levels in multiple tissues, such as adipose tissue, skeletal muscle, macrophages, and intestinal epithelium (colon). The PPARγ2 (NM015869.5; NP_056953.2) contains 505 amino acids and is predominantly expressed in WAT, BAT, and liver (13, 14). PPARγ3 (NM_001330615.4; NP_001317544.2) is expressed in macrophages, adipose tissue, and large intestine epithelium and has 278 amino acids (15). **Figures 1A, B** highlight the *PPARG1*, *PPARG2*, and *PPARG3* transcripts and PPARγ1, 2, and 3 isoform domains, respectively.

Both PPARγ1 and PPARγ2 isoforms have the intrinsic capacity to promote adipogenesis. However, recent studies show that PPARγ2 has a more decisive action on adipogenesis due to sensitivity to ligands and greater binding capacity to components of the mediator complex (16–18).

The PPARγ proteins have a molecular structure similar to that of other nuclear receptors and contain four domains: 1) N-terminus contains the Activation Function-1 (AF-1; also known as ligand-independent transactivation domain 1); 2) DNA binding domain (DBD); 3) HINGE, and 4) Ligand binding domain (LBD) located in the portion C-terminus. The AF-1 domain regulates ligand-independent transcriptional PPARγ activity, while the HINGE domain is involved in interactions with coactivators and corepressors. The DBD and LBD domains are the most essential and highly conserved among species. The DBD has the role of binding PPARγ to the promoter region of their target genes. The LBD domain is involved with ligand binding, the transactivation of many genes, and the transcriptional co-regulator interactions. The LBD is responsible for dimerization with the retinoid X receptor (RXR) and overlays the more powerful Activation Function-2 (AF-2) domain, which can be altered by the ligand binding (17, 19). AF-2 is the major transcriptional activation domain. It is essential for dimerization and regulates the ligand-dependent PPARγ transcriptional activity (1, 20–22). **Figures 1B** highlights the domains in PPARγ1, 2, and 3 isoforms. To better characterize the amino acid differences among the three main PPARγ isoforms, we performed an alignment to compare their protein sequences and domains, as shown in **Figure 1C**. These analyses were performed according to *Alvares et al.* (23). PPARγ2 has 30 additional amino acids in the N-terminus. Knockdown of *Pparg* in 3T3-L1 preadipocytes and *Pparg* null MEFs revealed that the PPARγ2 has a more potent role in adipocyte differentiation than the PPARγ1 isoform (17), indicating a crucial role of the longest PPARγ protein in the adipogenesis. However, how the N-terminus of PPARγ acts to promote adipogenesis remains an open question.

Despite being poorly conserved among species, the N-terminus portion showed an essential regulatory function in the action of PPARγ. The amino acids of the N-terminus domain have transcriptional activity when linked to a heterologous DNA-binding domain. Contradictorily, when this N-terminus region of PPARγ is deleted, this factor has greater transcriptional activity and more significant adipogenic action. This finding suggested that this N-terminus could also have some inhibitory function in the context of the holoreceptor, and a large part of this inhibitory action was linked to the phosphorylation of PPARγ by members of the MAP kinase family (20, 24). Furthermore, it was observed that the N-terminal domain influences the response to ligand binding of the LBD. Substitution of serine 112 by an aspartate residue inhibits ligand binding to the receptor (25).

To exert its biological action, PPARγ binds to members of the RXR family as an obligatory heterodimer at specific DNA binding sites, termed PPAR response elements (PPREs). The crystalline structure of the PPARγ-RXR heterodimer bounds to DNA in the presence of ligand results in a conformational change in the LBD and favors interaction with coactivator peptides (such as steroid receptor coactivators (SRCs), histone acetyltransferases (HATs), CBP and P300) and the Mediator complex, promoting transcription of PPARγ target genes, resulting in their physiological effects on adipogenesis and adipose tissue metabolism (12, 26).

## 4 PPARγ and adipogenesis

The PPARγ participates in adipogenesis during the differentiation of preadipocytes into adipocytes, playing a central role in this process (20). The evidence that PPARγ is the master regulator of adipogenesis is well established. *In vitro* and *in vivo* studies show a lack of matured adipocytes without PPARγ (27).

During the adipocyte differentiation process, PPARγ participates in a transcriptional cascade. Its activation promotes the induction of a variety of differentiation-dependent target genes, which play an essential role in the uptake and storage of triglycerides in the adipocyte (18, 28, 29).

**Figure 2:**
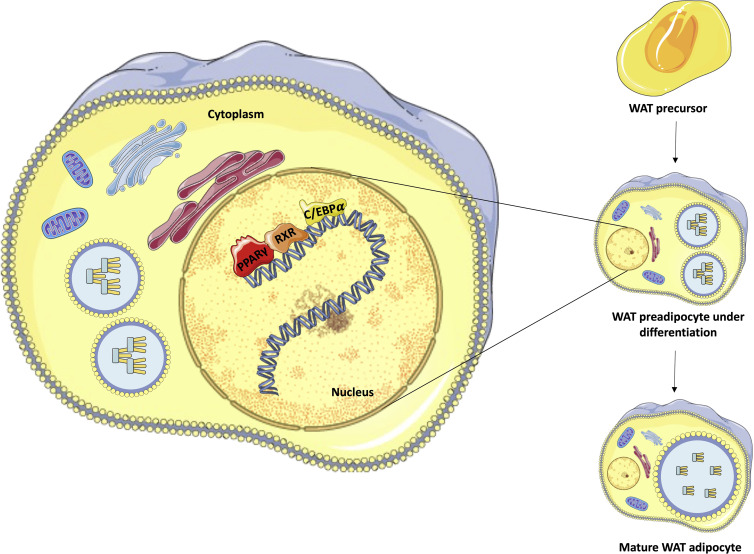
Representation of adipocyte differentiation control performed by PPARγ, C/EBP, and RXR. The figure emphasizes the process of differentiation of white adipocytes from pre-adipocytes, highlighting the transcriptional roles of PPARγ (red), C/EBP (orange), and RXR (yellow) in the nucleus of preadipocytes under differentiation to complete maturation. Own authorship using resources from SMART – Servier Medical Art.

After ligand activation, PPARγ induces many target genes involved in lipogenesis and adipogenesis and activates the expression of C/EBPα. This transcription factor can bind directly to the CEBP site in the PPARγ promoter, creating a stable, self-reinforcing regulatory loop (30, 31). After activation, PPARγ stimulates regulatory regions of a large number of genes that have essential roles in lipogenesis and insulin sensitivity, including *FABP4*, *PCK2, LPL, ADIPOQ, PLIN1*, and *SLC2A4* (which encode aP2, PEPCK, lipoprotein lipase, adiponectin, perilipin and Glut4 proteins, respectively), promoting the maturation of the adipocyte, which begins to capture and store lipids (**Figure 2**) (26, 28).

Likewise, the PPARγ is essential for the development and function of BAT. PPARγ ligands induce terminal differentiation of the brown preadipocyte HIB-1B cell line and stimulate the expression of UCP-1, a mitochondrial proton transporter that confers thermogenic properties to BAT (32).

## 5 Insulin sensitivity and PPARγ

**Figure 3:**
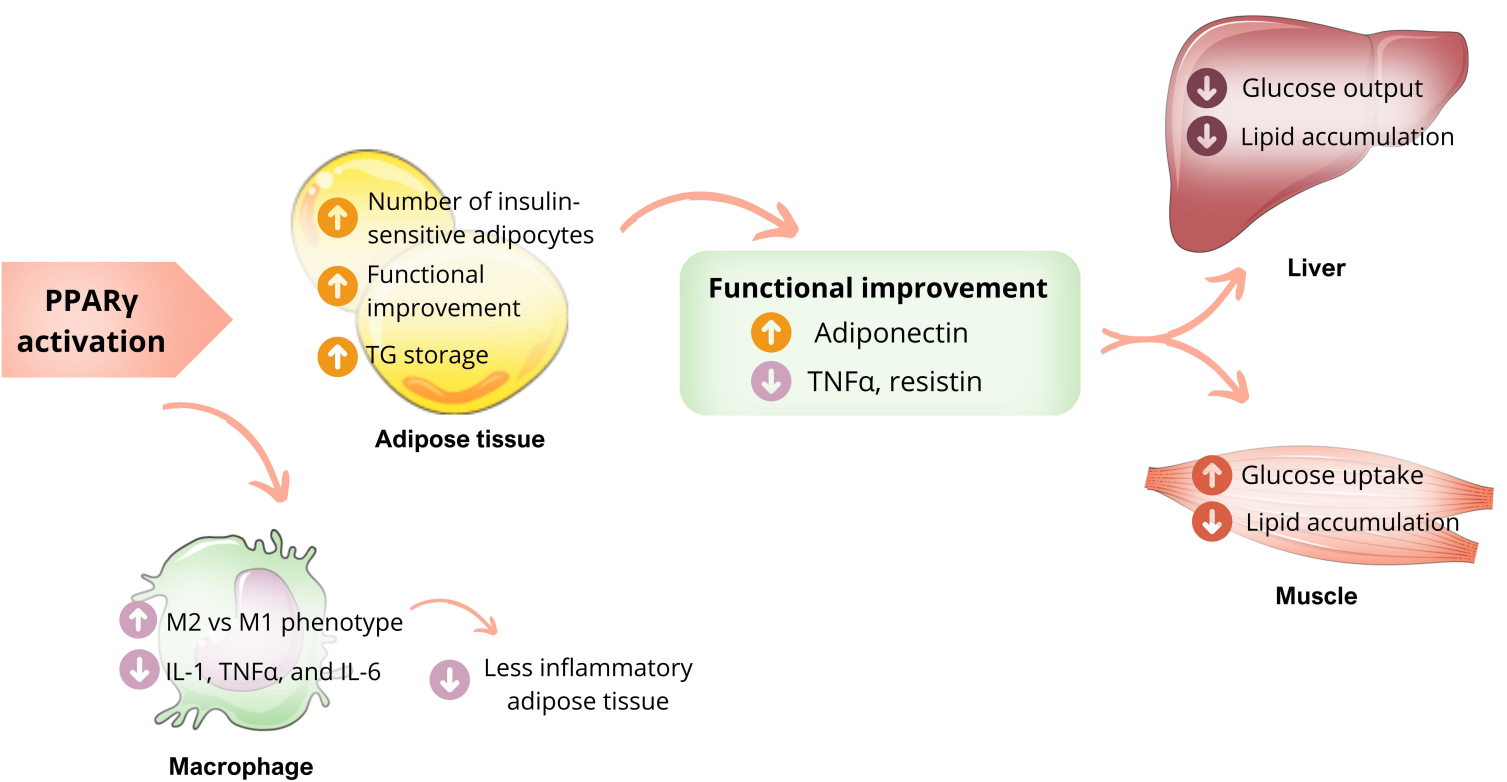
PPARγ actions and insulin sensibility. Activation of PPARγ in adipose tissue promotes the differentiation of pre-adipocytes into insulin-sensitive adipocytes, favoring the uptake of more lipids and influencing the production of adipokines, resulting in higher levels of adiponectin and reduced levels of TNF-α. These mechanisms benefit glucose metabolism, including less hepatic glucose production and more skeletal muscle glucose uptake, improving insulin sensibility. PPARγ activation also stimulates the transformation of macrophages into less inflammatory cells, thereby reducing macrophage infiltration into adipose tissue. Own authorship using resources from SMART – Servier Medical Art.

With the discovery of thiazolidinediones (TZDs) and their hypoglycemic action by improving IR, several studies were dedicated to understanding the role of PPARγ in insulin sensitivity. Activation of PPARγ by endogenous or synthetic ligands results in systemic insulin sensitization through complex mechanisms involving multiple organs. In adipose tissue, activated PPARγ promotes pre-adipocyte differentiation into insulin-sensitive adipocytes. This activation of PPARγ does not increase the size of adipocytes (hypertrophy); instead, it leads to the formation of smaller and more insulin-sensitive adipocytes, possibly due to *de novo* differentiation (12, 31). This process increases the capacity of WAT to store fatty acids (FA), reducing the ectopic concentration of FFA, whose accumulation leads to harmful effects on insulin action (33, 34). An additional but also important mechanism that favors insulin sensitivity is the functional improvement of adipose tissue after activation of PPARγ, which starts to produce more adiponectin, directly linked to insulin sensitivity (20). The adiponectin actions are already well established, improving muscle glucose uptake and reducing hepatic glucose production (**Figure 3**) (35). PPARγ activation by ligands in adipocytes is also associated with decreased levels of adipokines related to the IR onset, including tumor necrosis factor-alpha (TNFα) and resistin (36).

PPARγ plays a crucial role in promoting the alternative activation of macrophages, which results in less inflammatory adipose tissue (12). The relationship between macrophages and IR is already known. When macrophages infiltrate adipose tissue, they produce inflammatory cytokines such as IL-1, TNFα, and IL-6, which act on the insulin receptor. This causes the exchange of the phosphorylation residue to tyrosine by serine, resulting in less activation of the receptor and consequent IR. However, macrophages can also be activated alternatively (M2), producing arginase I (argl) and IL-10. These cytokines have less inflammatory power and less impact on the insulin receptor. PPARγ, especially the PPARγ3 isoform, plays a vital role in stimulating this alternative activation (M2), which configures the anti-inflammatory functions of this nuclear receptor (**Figure 3**) (15, 37).

## 6 *PPARG* pathogenic variants and metabolic diseases

Several pathogenic variants in the *PPARG* gene have been identified in the human population. Approximately 0.2% of the population presents missense variants of *PPARG*, but only 20% of these variants show functional impairments and are associated with metabolic commitments (38). The most common variant in the human *PPARG* gene is an alanine to proline substitution at position 12 in the PPARγ2 isoform (Pro12Ala) that has a variable physiological effect related to a decreased risk of type 2 diabetes mellitus (DM) (39).

In the general population, 1 in every 500 people carries a *PPARG* missense variant. However, only a small portion of them experience metabolic consequences that do not necessarily lead to FPLD3. After evaluating the FPLD3 clinical and phenotypic findings (**Table 1**), heterogeneity is observed among different *PPARG* pathogenic variants or members of the same family with similar pathogenic variants. This shows that the activity and expression of this gene are influenced by gene-gene and gene-environment interactions that determine the clinical consequences of genetic variants with significant genetic and phenotypic heterogeneity (38). Studies on mice indicate that a reduction in PPARγ activity up to 50% is still sufficient to maintain normal body composition. However, when PPARγ activity was reduced to 25%, it caused IR, decreased total body and fat mass, and dyslipidemia (66).

**Table 1:** Phenotype and clinical characteristics of the most frequent *PPARG* variants related to FPLD3.

41 *PPARG* variants (protein level)	Number of patients (Gender)	Age at genetic diagnosis	Age of onset FPLD3 (index case)	BMI kg/m²*	sWAT loss (sites)	Fat mass ratio (FMR)**	Hypertriglyceridemia	Diabetes (age dx)	Insulin therapy (u/day)	IR***	Hypertension	PCOS§	Pacreatitis	MAFLD♯	Reference
DBD DOMAIN – 16 variants															
**FS138X**	1(F)	37	17	33	Gluteal, lower limbs	NA	–	+ (13yr)	+ (100u/day)	+	+	NA	NA	NA	(40)
**Cys142Arg**	1(F)	41	34	30	Gluteal, limbs	1,5	+	+ (41yr)	NA	+	+	+	–	+	(41)
**Gly148Glu**	3 (F/F/M)^a^	18 (index case)	Puberty	NA	Gluteal, lower limbs	NA	+	–	–	+	NA	NA	–	+	(1)
**Tyr151Cys**	2(F)	61/34	43	23-24	NA	NA	+/+	+/+ (NA)	+ (142/125u/d)	+/+	+/+	-/+	+/+	NA	(42)
**Tyr151Cys**	1(F)	24	15	NA	Gluteal, lower limbs	NA	+	+ (22yr)	–	+	NA	NA	NA	NA	(43)
**Tyr151Cys**	4(F/M/F/F)	28 (index case)	15		Gluteal, lower limbs	NA	+/NA/+/+	+/NA/+/+	-/NA/+ (NA)/-	+/-/+/+	NA	+/-/+/+	NA	+/-/+/+	(43)
**Glu157Asp**	7 (F), 8 (M)	45 (32–55)	NA	NA	Extremities	NA	+ in 14 patients	+ in 8 patiens	+ in 6 patients (3 with > 100u/day)	+	+ in 11 patients	2 Females	+ in 7 patiens	+ in 9 patients	(44)
**Glu157Gly**	1(F)	64	29	19,7	Gluteal, upper and lower limbs	NA	+	+ (29yr)	+ (230u/day)	+	+	+	NA	+	(45)
**Cys159Tyr**	1(F)	42	35	24.2	Gluteal, lower limbs	1,58	+	+ (42yr)	NA	+	+	+	–	+	(41)
**Gly161Val**	1(F)	60	43	27	Gluteal, upper and lower limbs	1,47	+	+ (NA)	+ (160u/day)	+	+	NA	+	NA	(46)
**Arg164Trp**	1(F)^b^	30	Infance	21.2	Face, extremities, gluteal, back, abdomen	NA	+	+ (NA)	+ (100-300u/day)	+	–	NA	+ (13 episodes)	+	(47)
**Arg165Thr**	2(F)	44/22	22	23.8/26,5	Upper and lower limbs	2,23/1,78	+	+/+	+/+ (146u/day)	+/+	+/+ (severe)	NA	NA	+/+	(48)
**Leu178Pro**	1(F)	38	35	22.6	Upper and lower limbs	NA	+(severe)	+(35yr)	+(NA)	+	–	NA	–	+	(23)
**FS186X**	5(F), 2(M)	41 (21–71)	NA	NA	NA	NA	+ in 5 patients	+ in 4 patients	NA	+ in 5 patients	+ in 6 patients	NA	NA	NA	(49)
**Cys190Trp**	1(F)	31	19	30.5	Gluteal, lower limbs	NA	+	+ IGT (29)	–	+	+	+	–	+	(41)
**Cys190Ser**	3 (F/F/M)	26/36/60	26	29.8/28,3/29,8	Extremities in 3 patients	NA	+/+/+	+/+/+	+ (80u/d)/-/-	+/+/+	+/NA/NA	-/-	+/-/-	+/+/+	(50)
**Arg194Trp**	1(F)	31	19	25	Gluteal, extremities	NA	+	+ (15yr)	+ (300u/d)	+	+	+	+	NA	(51)
**Arg194Gln**	1(F)	46	24	24	Gluteal, limbs	NA	+	+ (NA)	NA	+	+	+	NA	+	(38)
HINGE – 4 variants															
**Met203Ile**	1(F)	17	10	26	Gluetal, limbs	1,25	+	+	NA	+	–	+	NA	+	(38)
**Arg212Gln**	1(F)	25	23	NA	Glutea, lower limbs	NA	+	+ IGT	–	+	+	+	–	+	(52)
**Arg212Trp**	1(F)	31	15	28	Gluteal, limbs	1,67	+ (mild)	+ (NA)	NA	+	+	+	NA	+	(38)
**Pro214Leu**	2(F/M)	34/32	28	23,1/26,7	Upper and lower limbs/-	1,70/1,73	+/+	-/-	-/-	+/+	-/-	NA	+/-	+/-	(23)
LBD DOMAIN – 21 variants															
**Ala261Glu**	2(F) (unrelated)	22/39	20/30	26/26	NA/NA	NA/NA	+/+	+/+	NA/NA	+/+	-/+	+/+	NA/NA	NA/NA	(53)
**Arg308Pro**	1(F)	16	16	23	NA	1,22	+	+	NA	+	–	+	NA	+	(38, 53)
**Phe310Ser**	1(F)	16	NA	17,5	Face, hips, limbs	NA	+	+ (16y)	+ (NA)	+	–	–	–	+	(54)
**Val318Met**	1(F)	16	15	25,6	NA	NA	+	+ (17yr)	–	+	+	+	NA	NA	(55)
**Leu339X**	2(M)	40/70	NA	28,6/23,9	Limbs (both)	1,72/NA	+/+	+ (NA)/-	+ (NA)/-	+/+	+/+	NA	NA	+/+	(48)
**FS343X**	1(F)	13	8	25,9	Gluteal, limbs	1,47	+	+ (8yr)	NA	+	–	+	–	+	(41)
**Lys347Thr**	2(F) 3(M)	61/40 38/54/29	NA	25,6/25,7 25,1/33/27	Limbs (all)	NA	+ (all)	+(36)/+(35) -/+(22)/+(21)	+(300u/d)/- -/+(220)/+	NA	+(All)	-/- NA	+/- -/+/+	+/+ NA/+/-	(56)
**Glu352Gln**	1(F)	26	NA	25	Lower limbs	NA	+ (mildly)	+ (13yr)	+ (200u/day)	+	–	NA	NA	+	(57)
**Ile354Val**	2 (F)	59/36	NA	30/23,4	Extremities	NA	+/-	+(58y)/+(17y)	NA	+/+	+/+	NA/-	NA	+/+	(58)
**Tyr355X**	2(F)	45/12	33	30/NA	Gluteal and limbs/no fat loss	NA	+/+	+(33y)/-	+ (2u/kg/d)/-	+/-	-/-	-/-	+/-	+/-	(59)
**Thr356Arg**	1(F)	19	19	34	Gluteal, lower limbs	1,47	+	+(NA)	NA	+	–	+	NA	+	(38)
**Arg385X**	1(F)	35	26	29,3	Gluteal, lower limbs	2,54	+ (severe)	+ (26yr)	NA	+	+	+	+	+	(41)
**Pro387Ser**	1(F)	13	8	20	Gluteal, limbs	NA	–	+ (NA)	NA	+	–	+	NA	+	(38)
**Phe388Leu**	2(F) 2(M)	46/22 71/39	NA	NA	Gluteal, lower limbs (all)	NA	+(all)	+/- +/-	-/- -/-	+/+ +/-	-/- +/-	+/- NA	NA	NA	(60)
**Ala417Val**	1(F)	40	39	36	Gluteal, lower limbs	NA	+	+(NA)	NA	+	–	+	NA	+	(38)
**Asp424Asn**	2(F)	14/36	NA	28,7/29,7	Limbs (all)	NA	+/+	-/-	-/-	+/+	+/+	NA/-	-/+	+/NA	(61)
**Arg425Cys**	1(F)	64	32	22,3	Limbs, face	NA	+	+(32y)	+ (NA)	+	+	+	NA	NA	(62)
**His449Leu**	3(F)^c^ 1(M)	23	16	23,6	Extremities	NA	+	+(22y)	+(22u/d)	+	–	+	NA	+	(63)
**Leu451Pro**	4(F)	NA	NA	NA	Limbs	NA	+(all)	+(NA) (all)	NA	+(all)	+(in 2 patients)	NA	NA	NA	(64)
**Thr468Lys**	1(F)	15	7	31	Gluteal, lower limbs	NA	+	+(NA)	NA	+	+	NA	NA	NA	(38)
**Pro495Leu**	1F/1M^c^ 1(F)	56 51	NA NA	24,9 18,5	NA Upper limbs and lower leg	NA NA	+ + (mild)	+(25y) +(41yr)	+(280u/d) -	+ +	+ -	NA NA	NA-	NA+	(55, 65)

NA denotes not available.

*The body-mass index (BMI) is the weight in kilograms divided by the square of the height in meters.

** Fat mass ratio (FMR) is the trunk fat % divided by the leg fat %. FMR>1.2 in women is consistent with lipodystrophy, although not diagnostic in itself.

***IR = Insulin Resistance: defined by the presence of acanthosis nigricans and/or HOMA-IR>3.0 and/or high fasting insulin levels.

♯ Metabolic associated fatty liver disease (MAFLD).

§Polycystic ovary syndrome (PCOS).

a Clinical data presented are from the index case. The second affected member was the mother, who presented fat loss, diabetes, and hypertriglyceridemia. The third member is her brother, who is 3 years old, and without clinical symptoms.

b This patient presented biallelic *PPARG* pathogenic variants (FS138X and Arg164Trp), leading to a phenotype of a generalized sWAT loss similar to CGL.

c Clinical data presented are from the index case.

Genetic lipodystrophies due to loss-of-function *PPARG* variants occur at a low frequency estimated at 1:100,000 individuals (67). These variants reduce the PPAR receptor activity and can lead to severe metabolic consequences associated with FPLD3. This disease has an autosomal dominant inheritance (all patients are heterozygous). It includes mainly amino acid substitutions (mainly in the DBD and LBD) and nonsense and frameshift mutations resulting in PPARγ inactivity (12).

As far as we know (based on the literature review carried out in PubMed, ClinVar, and The Human Gene Mutation Database), 44 *PPARG* pathogenic variants related to FPLD3 have been described until July 2024. These variants impair PPARγ transcriptional activity in several ways: some of the DBD variants show extreme dominant negative activity, suppressing the transcriptional activity of PPARγ; others cause an apparent impairment of transcriptional activity but do not show any dominant negative activity against the wild-type receptor. Likewise, variants affecting the LBD present dominant negative activity, even with little or no DNA binding activity (41). The clinical/phenotypic features of loss-of-function variants in the *PPARG* gene and FPLD3 will be discussed below.

## 7 Phenotype and clinical characteristics in PPARγ variants related to FPLD3

FPLD3 certainly impacts the quality and expectations of individuals with this condition. Unfortunately, the exact natural history of these syndromes is not well documented, making their diagnosis difficult and creating an obstacle in developing specific therapies for this disease (43).

Patients generally present a loss of adipose tissue in the hips and lower limbs, severe IR, diabetes, hyperglyceridemia, hypertension, hepatic steatosis, and, in women, polycystic ovary syndrome (PCOS) with symptoms of hyperandrogenism were found. Classically, FPLD3 is characterized by a milder loss of adipose tissue and a more severe metabolic condition when compared to FPLD2 (Dunnigan disease) (58). One possible explanation for this paradoxical finding is that patients harboring *PPARG* pathogenic variants may have fewer small, insulin-sensitive adipocytes, with the preservation of large adipocytes. This could explain why individuals with FPLD3 experience less severe loss of fat tissue (lipoatrophy) despite having higher insulin resistance than those with FPLD2 (68).

We have reviewed the clinical features presented by the index case and the affected family members in 41 out of 44 *PPARG* variants associated with FPLD3. The summarized data is available in **Table 2**, while detailed information about each specific variant is provided in **Table 1**. The other three pathogenic variants (Pro387Ser, Lys395Arg, and Gln438Pro) were initially described by SekizKardes et al. However, clinical data on affected patients are not available (69). Since the majority of *PPARG* variants reviewed here were previously described without considering the Human Genome Variation Society (HGVS) recommendations, we classified all reviewed *PPARG* variants according to HGVS (70), and their pathogenicity classification was made according to the American College of Medical Genetics and Genomics (ACMG) (71). We also used the MutationTaster and Mutalyzer tools to confirm the HGVS nomenclature (72, 73). These data were inserted in **Table 3**. This analysis was challenging since most manuscripts reviewed here did not include detailed information concerning the variants at the coding DNA and protein levels. Therefore, to classify all *PPARG* variants according to ACMG guidelines, we first used the MutationTaster tool to obtain the correct *PPARG* variant position at genomic DNA, cDNA, and protein levels for missense, nonsense, and the frameshift variants FS138X and FS343X. Then, the Mutalyzer tool was used to confirm the correct HGVS nomenclature. For the FS186X variant, the correct *PPARG* variant nomenclature was obtained using only the Mutalyzer tool since this frameshift variant could not be analyzed by the MutationTaster tool. We used the GRCh37 genome reference for all Mutalyzer tool analyses. For manuscripts that informed only the variant nomenclature at the protein level, the transcript and protein sequences were obtained from the National Center of Biotechnology Information (NCBI). For all analyses, we used the *PPARG* transcript sequence that encodes the biggest PPARγ isoform 2 (NC_000003.11; NM_015869.5; NP_056953.2). Then, after obtaining all appropriate nomenclatures, in silico predictive algorithms were applied, as recommended by ACMG guidelines. Here, we evaluated the pathogenicity of all *PPARG* missense and nonsense variants reviewed using CADD (Combined Annotation Dependent Depletion) v.1.7 (74) and REVEL (rare exome variant ensemble learner) tools (75). After these steps, we classified all *PPARG* variants reviewed according to ACMG guidelines (**Table 3**). More details were inserted in the footnote of **Table 3**.

**Table 2:** Overview of FPLD3 clinical characteristics related to 41 *PPARG* pathogenic variants.

	DBD domain + Hinge	LBD domain	Total
N° of pathogenic variants per domain	20	21	41
N° of patients described	50	41	91
Female/Male	37(F)/13(M)	32(F)/9(M)	69(F)/22(M)
Age of genetic diagnoses yr - μ	34(17-64)	36 (12-71)	33(12-71)
Age of onset FPLD3 yr - μ	23,5(10-43)	19(7-39)	21(7-43)
BMI kg/m^2^ - μ	26,6(19,7-30,5)	25,7(17,5-36,0)	26,0(17,5-30,5)
Hypertriglyceridemia - %	90%	94,5%	91,9%
Diabetes - %	72%	83,7%	77,0%
Age of onset diabetes yr – μ	29(13-42)	23,5(8-58)	25,5(8-58)
Hypertension - %	63,8%	54,0%	59,5%
PCOS - %*	57,6%	54,5%	56,2%
MAFLD - %**	81,0%	86,3%	87,5%

*Polycystic ovary syndrome (PCOS) - % calculate considered only the female population and excluded those with unavailable data.

^**^Metabolic-Dysfunction-Associated Fatty Liver Disease (MAFLD) - Patients with unavailable data (31 patients) were excluded from the % calculation.

**Table 3:** Deleteriousness predictions of all 41 previously published *PPARG* variants according to the American College of Medical Genetics and Genomics (ACMG).

41 *PPARG* pathogenic variants (protein level) (Original nomenclature but considering PPARγ isoform 2)^a^	Nomenclature of *PPARG* pathogenic variants according to HGVS (at DNA and protein levels)^b^	Reference	CADD	REVEL	ACMG classification criteria
DBD DOMAIN – 16 variants					
**FS138X**	c.413_416delAATG p.(Glu138Valfs*31)	(40)	–	–	Pathogenic: PVS1, PM1, PM2, PM4, PP2, and PP3
**Cys142Arg**	c.424T>C p.Cys142Arg	(41)	24.5	0.95	Likely pathogenic: PS3, PM1, PM2, PP2, and PP3
**Gly148Glu**	c.443G>A p.(Gly148Glu)	(1)	25.1	0.94	Likely pathogenic: PM1, PM2, PP2, and PP3
**Tyr151Cys**	c.452A>G p.Tyr151Cys	(42)	26.4	0.97	Likely pathogenic: PS3, PM1, PM2, PP2, and PP3
**Glu157Asp**	c.471A>C p.Glu157Asp	(44)	24.2	0.89	Pathogenic: PS3, PM1, PM2, PM5, PP2, and PP3
**Glu157Gly**	c.470A>G p.(Glu157Gly)	(45)	29	0.97	Likely pathogenic: PM1, PM2, PM5, PP2, and PP3
**Cys159Tyr**	c.476G>A p.Cys159Tyr	(41)	28.9	0.97	Likely pathogenic: PS3, PM1, PM2, PP2, and PP3
**Gly161Val**	c.482G>T p.(Gly161Val)	(46)	32	0.94	Likely pathogenic: PM1, PM2, PP2, and PP3
FS138X and Arg164Trp*	c.413_416delAATG and c.490C>T p.(Glu138Valfs*31) and p.(Arg164Trp)*	(47)	- and 32	- and 0.97	Pathogenic: PVS1, PM1, PM2, PM4, PP2, and PP3 and Pathogenic: PVS1, PM1, PM2, PM4, PP2, and PP3
**Arg165Thr**	c.494G>C p.Arg165Thr	(48)	27.9	0.97	Likely pathogenic: PS3, PM1, PM2, PP2, and PP3
**Leu178Pro**	c.533T>C p.(Leu178Pro)	(23)	24.7	0.73	Likely pathogenic: PM1, PM2, PP2, PP3, and PP4.
**FS186X**	c.554_556del_insT p.Lys185Metfs*2	(49)	–	–	Pathogenic: PVS1, PS3, PM1, PM2, PM4, PP2, and PP3
**Cys190Trp**	c.570T>G p.Cys190Trp	(41)	25.8	0.92	Likely pathogenic: PS3, PM1, PM5, PP2, and PP3
**Cys190Ser**	c.568T>A p.Cys190Ser	(50)	26.7	0.98	Likely pathogenic: PS3, PM1, PM5, PP2, and PP3
**Arg194Trp**	c.580C>T p.Arg194Trp	(51)	31	0.95	Likely pathogenic: PS3, PM1, PM5, PP2, and PP3
**Arg194Gln**	c.581G>A p.Arg194Gln	(38)	32	0.89	Likely pathogenic: PS3, PM1, PM5, PP2, and PP3
HINGE – 4 variants					
Met203Ile^c^	c.609G>A, c.609G>C, or c.609G>T p.Met203Ile	(38)	27 for the three possibilities	0.94 for the three possibilities	Likely pathogenic: PS3, PM1, PM2, PP2, and PP3
**Arg212Gln**	c.635G>A p.(Arg212Gln)	(52)	32	0.91	Likely pathogenic: PS3, PM1, PM2, PM5, PP2, and PP3
**Arg212Trp**	c.634C>T p.Arg212Trp	(38)	32	0.90	Likely pathogenic: PS3, PM1, PM2, PM5, PP2, and PP3
**Pro214Leu**	c.641C>T p.(Pro214Leu)	(23)	27	0.71	Likely pathogenic: PM1, PM2, PP1, PP2, PP3, PP4, and PP5.
LBD DOMAIN – 21 variants					
**Ala261Glu**	c.782C>A p.Ala261Glu	(53)	29.5	0.73	Likely pathogenic: PS3, PM1, PM2, PP2, and PP3
**Arg308Pro**	c.923G>C p.Arg308Pro	(38, 53)	27.6	0.56	Likely pathogenic: PS3, PM1, PM2, PP2, and PP3
**Phe310Ser**	c.929T>C p.(Phe310Ser)	(54)	31	0.79	Likely pathogenic: PM1, PM2, PP2, and PP3
**Val318Met**	c.952G>A p.Val318Met	(55)	28.8	0.53	Likely pathogenic: PS3, PM1, PM2, PP2, and PP3
**Leu339X**	c.1016T>A p.Leu339*	(48)	39	–	Pathogenic: PVS1, PS3, PM1, PM2, PM4, PP2, and PP3
**FS343X**	c.1024delC p.Gln342Lysfs*2	(41)	–	–	Pathogenic: PVS1, PM1, PM2, PS3, and PM4
**Lys347Thr**	c.1040A>C p.(Lys347Thr)	(56)	27.1	0.89	Likely pathogenic: PM1, PM2, PP2, and PP3
**Glu352Gln**	c.1054G>C p.(Glu352Gln)	(57)	27.9	0.79	Likely pathogenic: PM1, PM2, PP2, and PP3
**Ile354Val**	c.1060A>G p.Ile354Val	(58)	22.9	0.18	Likely pathogenic: PS3, PM1, PM2, PP2, and PP3
**Tyr355X**	c.1065C>G p. Tyr355*	(59)	36	–	Pathogenic: PVS1, PS3, PM1, PM2, PM4, PP2, and PP3
**Thr356Arg**	c.1067C>G p. Thr356Arg	(38)	26.2	0.68	Likely pathogenic: PS3, PM1, PM2, PP2, and PP3
**Arg385X**	c.1153C>T p.Arg385*	(41)	36	–	Pathogenic: PVS1, PS3, PM1, PM2, and PM4
**Pro387Ser**	c.1159C>T p. Pro387Ser	(38)	28.4	0.83	Likely pathogenic: PS3, PM1, PM2, PP2, and PP3
Phe388Leu^d^	c.1164T>A or c.1164 T>G p.Phe388Leu	(60)	25.9 or 25.7	0.85 for both	Likely pathogenic: PS3, PM1, PM2, PP2, and PP3
**Ala417Val**	c.1250C>T p.Ala417Val	(38)	26	0.8	Likely pathogenic: PS3, PM1, PM2, PP2, and PP3
**Asp424Asn**	c.1270G>A p. Asp424Asn	(61)	34	0.75	Likely pathogenic: PS3, PM1, PM2, PP2, and PP3
**Arg425Cys**	c.1273C>T p.Arg425Cys	(62)	27.7	0.78	Likely pathogenic: PS3, PM1, PM2, PP2, and PP3
His449Leu^e^	c.1430A>T p.(His447L)	(63)	27.9	0.916	Likely pathogenic: PM1, PM2, PP2, and PP3
**Leu451Pro**	c.1352T>C p.Leu451Pro	(64)	24.3	0.58	Likely pathogenic: PS3, PM1, PM2, PP2, and PP3
**Thr468Lys**	c.1403C>A p.Thr468Lys	(38)	27.8	0.59	Likely pathogenic: PS3, PM1, PM2, PP2, and PP3
**Pro495Leu**	c.1484C>T p.Pro495Leu	(55, 65)	29.6	0.9	Likely pathogenic: PS3, PM1, PM2, PP2, and PP3

a In order to categorize the *PPARG* pathogenic variants, whether isoform 1 (NP_001341595.2) was used in the reviewed manuscripts to obtain the variant nomenclature, we converted the nomenclature according to PPARγ isoform 2 (NP_056953.2).

b The *PPARG* transcript variant used to obtain the HGVS nomenclature was NM_015869.5, corresponding to PPARγ isoform 2.

c Since the amino acid isoleucine (Ile) is encoded by three different codons (ATA, ATC, and ATT) and the original manuscript did not inform the *PPARG* variant at the DNA level, we inserted the three HGVS nomenclatures.

d Since the amino acid leucine (leu) is encoded by two different codons (TTG and TTA) similar to methionine (Met) and the original manuscript did not inform the *PPARG* variant at the DNA level, we inserted both HGVS nomenclatures.

e This nomenclature in the original manuscript was based on isoform 1. Here we considered isoform 2.

All in silico predictive algorithms applied here follow the ACMG standards and guidelines (72). ACMG pathogenic criteria include: i) PVS, ii) PS, iii) PM, and iv) PP, which mean: i) pathogenic very strong, ii) pathogenic strong, iii) moderate, and iv) supporting pathogenicity, respectively.

Pathogenic variants: (i) 1 Very strong (PVS1) and (a) ≥1 Strong (PS1–PS4) or (b) ≥2 Moderate (PM1–PM6) or (c) 1 Moderate (PM1–PM6) and 1 supporting (PP1–PP5) or (d) ≥2 Supporting (PP1–PP5) or (ii) ≥2 Strong (PS1–PS4) or (iii) 1 Strong (PS1–PS4) and (a) ≥3 Moderate (PM1–PM6) or (b) 2 Moderate (PM1–PM6) and ≥2 Supporting (PP1–PP5) or (c)1 Moderate (PM1–PM6) and ≥4 supporting (PP1–PP5).

Likely pathogenic variants: (i) 1 Very strong (PVS1) and 1 moderate (PM1–PM6) or (ii) 1 Strong (PS1–PS4) and 1–2 moderate (PM1–PM6) or (iii) 1 Strong (PS1–PS4) and ≥2 supporting (PP1–PP5) or (iv) ≥3 Moderate (PM1–PM6) or (v) 2 Moderate (PM1–PM6) and ≥2 supporting (PP1–PP5).

CADD (Combined Annotation Dependent Depletion) v.1.7 (74). REVEL (rare exome variant ensemble learner) (75).

The CADD score above 20 indicates a variant predicted to be among the 1.0% most deleterious possible changes in the human genome.

The score for REVEL ranges from 0 to 1. Higher scores indicate a greater likelihood of the variant being disease-causing.

*This subject presents a phenotype of a generalized sWAT loss similar to CGL.

The nomenclature of all *PPARG* variants was updated according to HGVS (Human Genome Variation Society). All HGVS nomenclatures were based on transcript 2 (NM_015869.5), which encodes the biggest PPARγ isoform 2.

Parentheses were included in *PPARG* pathogenic variants at the protein level with no experimental data confirming the protein change.

Cases of FPLD3 usually begin to show clinical manifestations at puberty or early adulthood, and females are most affected (1). Of the 91 cases described, 69 (75.8%) were women. The patients started experiencing symptoms around age 21 (**Table 2**). The average age of onset of FPLD3 symptoms is around 20 years, similar to other FPLD, but there is a significant variability in onset among the patients listed, ranging from 7 to 43 years. One limitation in analyzing these data is that 43 out of the 91 patients listed did not have information about the onset of symptoms or perception of lipoatrophy in the limbs. When analyzing data from patients with FPLD 2, it is observed that the onset of symptoms occurs during puberty in women and later in men, with few cases presenting in childhood or senescence (76).

The loss of WAT tends to be variable and mainly affects the lower limbs and gluteal, but it can also extend to other areas, such as the upper limbs and face. They also show a specific WAT accumulation in some areas, which is variable in these patients. Some sWAT accumulate fat in the face, trunk, back, and abdomen, while others do not (45, 54, 62). Some FPLD3 patients may exhibit a more subtle loss of sWAT, while others may not experience any reduction at all (59).

Biallelic *PPARG* variants (compound heterozygous) were related to a congenital generalized lipodystrophy phenotype, emphasizing the genetic heterogeneity of congenital lipodystrophies. This is evident when observing a 37-year-old woman carrying the FS138X pathogenic variant who presented a loss of adipose tissue in the gluteal region and lower limbs but accumulation in the back, periscapular region, abdomen, and visceral fat (40). On the other hand, a 30-year-old woman who carried biallelic pathogenic variants (Arg164Trp and FS138X) in heterozygosity showed a loss of WAT on the face, lower limbs, and buttocks since childhood. It progressed to the upper limbs, back, and abdomen in adulthood, resembling the phenotypic pattern of generalized congenital lipodystrophies (CGL) (47). This patient’s case was the first one correlating distinct heterozygous *PPARG* variants with the CGL phenotype, associated with a pathogenic variant that had not yet been reported in the literature (Arg164Trp).

Objective assessment of fat distribution can be done using Computed Tomography (CT), Magnetic Resonance Imaging (MRI), and Dual-Energy X-ray Absorptiometry (DXA). DXA is more clinically applicable among these due to its better accessibility and lower cost. The Fat Mass Ratio (FMR - the ratio between truncal and lower limb fat) can be evaluated by DXA and has been suggested as a tool for identifying partial lipodystrophy. An FMR value greater than 1.2 may indicate a lipoatrophic pattern suggestive of partial lipodystrophy, although it is not diagnostic (77). In our analysis, 12 FPLD3 patients had FMR values available, all of which were greater than 1.2 (ranging from 1.22 to 2.54), underlining the usefulness of this parameter as an additional measure for diagnosing FPLD3 (**Table 1**).

The development of metabolic disorders associated with lipodystrophies is linked to the inability to maintain adequate fat storage in sWAT and impaired postprandial lipid buffering (1). When the capacity for sWAT expansion is impaired, fat is relocated in non-adipose organs, such as the liver, skeletal muscle, and pancreas (78). The dysfunctional adipocytes develop lipotoxicity, macrophage infiltration, mitochondrial dysfunction, and oxidative stress (8). The lipotoxicity alters insulin receptor signaling pathways, causing severe IR and abnormal metabolism of lipids and glucose (79). Further, excessive levels of inflammatory adipokines and cytokines secreted by visceral AT induce the accumulation of TG and FFA in ectopic sites (78, 80).

FPLD3 subjects present a more severe IR, and the extent of the change in adipose tissue is greater. Insulin resistance can be identified through clinical signs such as acanthosis nigricans and acrochordons. These conditions occur due to high insulin levels in keratinocytes and fibroblasts from the skin, leading to hyperkeratosis and skin hyperpigmentation, especially in skin fold areas. It’s important to note that while acanthosis nigricans is common in patients with FPLD3, acrochordons are not typically described in this group. In contrast, this condition has been observed in other lipodystrophies, such as CGL, but only in a minority of cases (4). Fasting blood glucose and insulin levels can be considered to assess IR biochemically, along with calculating the Homeostasis Model Assessment-Insulin Resistance (HOMA-IR) index (1). In our review of 91 patients, 84 showed clinical or biochemical signs of insulin resistance, indicating its significant role in developing metabolic complications of FPLD3.

As a result of this process, patients tend to develop DM in early adulthood. These patients typically present with difficult-to-control hyperglycemia and require high-dose insulin therapy due to the severity of IR. Many patients suffer from microvascular and macrovascular complications due to poor glycemic control (40, 42, 45). Previous studies have shown that FPLD3 patients are more likely to develop DM than those with FPLD2, with 72% of the patients experiencing it, while the prevalence of diabetes in Dunnigan disease ranged from 28% to 51% (68, 76). Our analysis confirms this, as 77% of the patients we reviewed had DM. The median age at which this condition appeared was 25.5 years. A wide variation in the onset of diabetes was observed in this analysis (ranging from 8 to 58 years). In many cases, this data was unavailable (42 cases out of 67 patients with DM). Among the available data, 24 patients were using insulin therapy, the majority with doses greater than 100 U/day or 2U/kg/day, and some requiring up to 5U/kg/day.

HTG is a metabolic change that occurs early on and is considered a lipid indicator of ongoing lipodystrophy. When WAT deposits are reduced due to lipoatrophy, TG present in circulating lipoproteins, chylomicrons, and VLDL can only be partially stored in these deposits. Another contributing factor is increased VLDL synthesis due to hepatic steatosis, which is also found in these patients. These mechanisms likely lead to an increase in circulating TG levels (81).

HTG is generally severe and can be accompanied by eruptive xanthomas and lead to complications such as acute pancreatitis. Triglyceride levels can be two to three times higher in women with FPLD compared to men (1). Patients with FPLD3 typically have more severe HTG and a higher risk of acute pancreatitis than those with FPLD2 (1). Among patients with FPLD3, 91.9% had HTG, with only three (Arg212Trp, Glu352Gln, Pro496Leu) having slightly increased TG levels (**Table 2**). Although most patients with FPLD3 present with moderate or severe HTG, these two cases with mild HTG highlight the phenotypic heterogeneity observed in lipodystrophies. When comparing the pathogenic variants Arg212Trp and Arg212Gln (located in the same position of the gene), a phenotypic difference is observed concerning the intensity of HTG and the age at which fatty tissue loss begins (earlier in the Arg212Trp variant), which highlights the possibility of gene-gene interactions and gene-environment. Acute pancreatitis was observed in 16 patients, and one of them had 13 episodes of pancreatitis. Seven distinct variants presented a phenotype that included eruptive xanthomas (41, 42, 47, 52, 59).

Due to insulin resistance and HTG, patients often experience metabolic-associated fatty liver disease (MAFLD), which can cause hepatomegaly and liver cirrhosis (58, 72). In this review, 87,5% of the patients for whom data was available developed this condition. Thirty-one patients did not provide any information about MAFLD.

PPARγ regulates vascular tone and blood pressure and is expressed in many vascular system components (endothelial and smooth muscle cells). Loss-of-function variants are associated with hypertension, which is generally severe and has an early onset (82). Initially, two cases of distinct mutations in the *PPARG* gene (Val318Met and Pro495Leu) were described with severe and difficult-to-control arterial hypertension, which appeared around 30 years of age (55). Patients with different variants (Arg165Thr and Leu339X) had severe hypertension with no systemic renin-angiotensin system (RAS) alterations. However, components of the cellular RAS were markedly overexpressed and activated in fibroblasts and peripheral blood mononuclear cells (PBMCs) issued from 4 patients with this variant (48). Patients’ cells exhibited increased levels of angiotensin II receptor 1 (AT1R), renin, and angiotensinogen (AGT), with overactivation of angiotensin II signaling and oxidative stress and inflammation. These findings suggest that severe hypertension, which is a peculiar feature of patients with FPLD3, might be linked to tissue RAS overactivation resulting from PPARγ dysfunction (48).

It is important to note that hypertension, which is one of the typical clinical symptoms of FPLD3 that we have studied, can manifest differently among patients. In total, 59,5% of patients experience hypertension, with a higher percentage of patients affected among those with a *PPARG* variant in the DBD (63,8%) compared to those with a variant in the LDB (54,0%) (**Table 2**).

Women with FPLD3 often experience changes in their reproductive system, such as menstrual irregularity, hirsutism, infertility, and polycystic ovary syndrome (PCOS) (8). These changes are related to IR, which can cause high levels of circulating insulin that affect the ovaries. This can lead to overproduction of androgens by theca cells and interfere with follicular stimulation and ovulation (38, 43, 53). After analyzing 39 mutations, researchers found that PCOS was present in 56.2% of women, usually accompanied by irregular menstrual cycles and hirsutism.

Different *PPARG* pathogenic variants can result in similar physical and metabolic characteristics mentioned above, but this is not always true. Even though two patients have similar or distinct pathogenic variants in the *PPARG* gene, they can exhibit different physical characteristics and clinical symptoms. This is because gene-gene and gene-environment interactions can contribute to phenotypic and clinical heterogeneity. The specific clinical findings related to each PPARG pathogenic variant are detailed in **Table 1**. Therefore, being aware of the critical clinical presentations associated with FPLD can help to enhance understanding of the FPLD disease and prevent misdiagnosis (3).

Some patients with *PPARG* pathogenic variants have exhibited clinical findings that are not typically associated with FPLD3. For instance, a patient with a variant in the DBD domain (FS138X) had bilateral cataracts and bilateral hearing deficits (40). Additionally, two patients with different variants (Gly148Glu and Phe310Ser) reported hypothyroidism (1, 54). Furthermore, two men with the Leu339X variant experienced psoriasis (48). After reviewing the literature, it was observed that there is an association of PPAR with thyroid carcinoma but not with hypothyroidism (83).

To date, no association between FPLD3 and cataracts has been observed in the literature. There is only one reported case of congenital, autosomal dominant, partial lipodystrophy. It was associated with congenital cataracts and spinal cord and cerebellar dysfunction. However, the specific pathogenic variant associated with this phenotype was not described (variants in *LMNA* or *BSCL2* genes were ruled out) (84).

Although many studies have evaluated the functional properties of PPARs in the eye and discovered fundamental PPAR mechanisms in the retina and cornea, PPARγ has not yet been associated with changes in the iris. PPARγ and PPARα are well established in their functions in ocular homeostasis regarding neuroprotection, neovascularization, and inflammation (85).

The literature contains no data supporting the connection between FPLD3 and psoriasis. However, some studies suggest the involvement of PPARγ in developing this skin condition, highlighting its direct effects on keratinocytes and immune cells. In psoriasis, the activation of PPARγ regulates the inflammatory response by reducing the expression and suppressing the genes of adhesion molecules. Additionally, the activation of PPARγ hinders the differentiation of Th CD4+ cells into Th17 cells. Some small-scale studies have shown improved skin symptoms after using pioglitazone in a limited number of patients (86).

Until now, there is no cure for FPLD3. Therapeutic approaches should be directed towards the associated comorbidities. This treatment is challenging and requires the combination of several strategies, such as lifestyle modifications and intensive treatment for DM and dyslipidemia (8). Lifestyle changes include physical exercise and a balanced diet containing approximately 50–60% of carbohydrates, 20–30% fat, and 20% protein (87). Due to the syndrome’s rarity, the evidence supporting pharmacological treatment is primarily based on expert opinion, case reports, or case series (88).

The initial management of hyperglycemia can benefit from insulin sensitizers like metformin and thiazolidinediones (TZDs), which are oral hypoglycemic options. TZDs medications can help manage partial lipodystrophies by stimulating the action of PPARγ to form sWAT and improve insulin sensitivity. There are reports of isolated cases using thiazolidinediones (TZD) in FPLD3 but with variable results. Pioglitazone showed favorable and sustained results in improving glycemic control and dyslipidemia in women carrying Tyr355X, His449Leu, Arg308Leu, and Phe310Ser pathogenic variants. These women initially had mild metabolic changes, except for the last two, who had more severe metabolic issues (53, 54, 59, 63). However, a different response was observed using rosiglitazone in patients with severe metabolic profiles who carried the *PPARG* pathogenic variants Pro495Leu, Val318Met, and Ala261Glu. In these cases, there was a slight and non-sustained improvement in blood glucose levels and serious adverse effects of the therapy. One possible explanation for this difference in response is the effectiveness of the medication. Pioglitazone is more effective than rosiglitazone in reducing the metabolic profile and improving fat distribution in animal models and clinical trials with DM2 patients (89–92). Another hypothesis suggests that the site of the *PPARG* pathogenic variant may interfere with its responsiveness to endogenous or synthetic ligands. In a study with structural modeling of *PPARG* pathogenic variants (Arg308Leu and Ala261Glu), it was found that the site of these variants interferes with the response to the endogenous ligand while maintaining a full transcriptional response to synthetic ligands, such as pioglitazone (Arg308Leu) and rosiglitazone (Ala261Glu). In the latter case, there was a metabolic improvement in clinical analysis, but serious adverse effects to the rosiglitazone were observed, resulting in treatment interruption (53, 93).

The sodium-glucose cotransporter 2 (SGLT2) inhibitors are essential for treating diabetes in lipodystrophic patients due to their insulin-independent effect and notable cardio-renal benefits. Bansal et al. analyzed the efficacy and safety of SGLT2 inhibitors in a cohort of 12 patients with partial lipodystrophy (4 with variants in *LMNA*, 1 in *PPARG*, 1 in *PCYT1A*, and 6 with unknown mutations). They found a significant reduction in HbA1c and blood pressure in patients using these medications. The most reported adverse effects included fungal infections, urinary infections, and limb pain. Serious adverse effects with diabetic ketoacidosis occurred in only one patient who was not compliant with insulin therapy (94).

Glucagon-like peptide 1 receptor agonists (GLP-1RA) have shown positive results in treating patients with FPLD. In a recent retrospective analysis, 13 patients with FPLD type 1 and 1 patient with FPLD type 2 were treated with GLP-1RA, and the metabolic effects of this treatment were observed by comparing the results before and six months after starting this drug. The treatment with GLP-1RA significantly reduced weight, BMI, HbA1c, and fasting glucose levels in FPLD patients. Additionally, triglyceride levels decreased from 334 ± 170 mg/dL before GLP-1RA treatment to 256 ± 82 mg/dL after six months (95).

Due to the severity of diabetes, insulin therapy is typically required for treating patients with FPLD. These patients often need high insulin doses, and in this situation, U500 insulins may be beneficial (8).

Metreleptin is a recombinant human leptin analog used as a specific therapy to manage human lipodystrophies. A study was conducted on seven patients with FPLD3 (Arg425Cys, Arg194Gln, Pro495Leu, Pro387Ser, Lys395Arg, and Gln438Pro pathogenic variants) who were treated with metreleptin for 13 months. The study showed that metreleptin improved glycemic control, as evidenced by decreased glycated hemoglobin and fasting glucose. The reduction in triglycerides in patients with *PPARG* pathogenic variants depended on the initial triglyceride value: four patients with baseline serum triglyceride levels >500 mg/dL were classified as metreleptin responders, whereas only one of three patients with baseline triglyceride levels <500 mg/dL was a responder (69).

## 8 Conclusion

Patients with *PPARG* loss-of-function variants display clinical symptoms that reflect the impact of this protein on the development and functioning of adipose tissue. PPARy is a significant regulator of adipogenesis and insulin response. The several pathogenic variants found in the *PPARG* gene leading to a classic pattern of FPLD3 show heterogeneity at the allelic level. Patients with the same pathogenic variant present some distinct clinical characteristics, suggesting heterogeneity at a clinical and phenotypic level. These interactions arise from gene-gene and gene-environment interactions. Understanding these characteristics will help in diagnosing this rare but underdiagnosed disease and can lead to more precise therapeutic interventions for patients.
