# Corrigendum: Familial partial lipodystrophy resulting from loss-of-function PPARγ pathogenic variants: phenotypic, clinical, and genetic features

**DOI:** 10.3389/fendo.2024.1508146

**Published:** 2024-12-03

**Authors:** Reivla Marques Vasconcelos Soares, Monique Alvares da Silva, Julliane Tamara Araújo de Melo Campos, Josivan Gomes Lima

**Affiliations:** ^1^ Department of Clinical Medicine, Hospital Universitaírio Onofre Lopes (HUOL), Federal University of Rio Grande do Norte (UFRN), Natal, RN, Brazil; ^2^ Molecular Biology and Genomics Laboratory, Federal University of Rio Grande do Norte (UFRN), Natal, RN, Brazil; ^3^ Department of Morphology (DMOR), Federal University of Rio Grande do Norte (UFRN), Natal, RN, Brazil

**Keywords:** PPAR gamma, adipose tissue, genetic lipodystrophy, insulin resistance, diabetes mellitus

In the published article, there was an error in the **Funding** statement. The **Funding** statement was displayed as “The author(s) declare financial support was received for the research, authorship, and/or publication of this article. This study was financed in part by the Coordenação de Aperfeiçoamento de Pessoal de Nível Superior - Brasil (CAPES) - Finance Code 001”. The correct **Funding** statement appears below.

“The authors declare that financial support was received for manuscript processing charges and the open access fee from Chiesi Global Rare Diseases (GRD). Chiesi GRD was not involved in the design, data collection, interpretation or decision to publish this manuscript.”

The authors apologize for this error and state that this does not change the scientific conclusions of the article in any way. The original article has been updated.

